# Differential CD4+ T-Cell Cytokine and Cytotoxic Responses Between Reactivation and Latent Phases of Herpes Zoster Infection

**DOI:** 10.20411/pai.v7i2.560

**Published:** 2023-02-17

**Authors:** Wenjie Jin, Mike Fang, Ismail Sayin, Carson Smith, Jeffrey L. Hunter, Brian Richardson, Jackelyn B. Golden, Christopher Haley, Kenneth E. Schmader, Michael R. Betts, Stephen K. Tyring, Cheryl M. Cameron, Mark J. Cameron, David H. Canaday

**Affiliations:** 1 Division of Infectious Diseases Case Western Reserve University, Cleveland, OH; 2 Division of Infectious Diseases and Geriatric Research, Education and Clinical Center (GRECC), Cleveland Veterans Administration Medical Center, Cleveland, OH; 3 Department of Population and Quantitative Health Sciences, Case Western Reserve University, Cleveland, OH; 4 Department of Family Medicine, Ohio University, Athens, OH; 5 Center for Clinical Studies and Department of Dermatology, University of Texas Health Science Center, McGovern School of Medicine, Houston, TX; 6 Division of Geriatrics, Duke University Medical Center and GRECC, Durham Veterans Affairs Medical Center, Durham, NC; 7 Department of Microbiology, University of Pennsylvania Perelman School of Medicine, Philadelphia, PA; 8 Department of Nutrition, Case Western Reserve University, Cleveland, OH

**Keywords:** VZV, Herpes Zoster, T-cell polyfunction, CD4 T cells, transcriptomic

## Abstract

**Background:**

CD4+ T cells are a critical component of effective immune responses to varicella zoster virus (VZV), but their functional properties during the reactivation acute vs latent phase of infection remain poorly defined.

**Methods:**

Here we assessed the functional and transcriptomic properties of peripheral blood CD4+ T cells in persons with acute herpes zoster (HZ) compared to those with a prior history of HZ infection using multicolor flow cytometry and RNA sequencing.

**Results:**

We found significant differences between the polyfunctionality of VZV-specific total memory, effector memory, and central memory CD4+ T cells in acute vs prior HZ. VZV-specific CD4+ memory T-cell responses in acute HZ reactivation had higher frequencies of IFN-γ and IL-2 producing cells compared to those with prior HZ. In addition, cytotoxic markers were higher in VZV-specific CD4+ T cells than non-VZV-specific cells. Transcriptomic analysis of *ex vivo* total memory CD4+ T cells from these individuals showed differential regulation of T-cell survival and differentiation pathways, including TCR, cytotoxic T lymphocytes (CTL), T helper, inflammation, and MTOR signaling pathways. These gene signatures correlated with the frequency of IFN-γ and IL-2 producing cells responding to VZV.

**Conclusions:**

In summary, VZV-specific CD4+ T cells from acute HZ individuals had unique functional and transcriptomic features, and VZV-specific CD4+ T cells as a group had a higher expression of cytotoxic molecules including Perforin, Granzyme-B, and CD107a.

## INTRODUCTION

VZV is a common human alphaherpesvirus [1, 2] that causes varicella (chickenpox) during primary infection. Following the acute phase, VZV establishes a latent infection in the dorsal root and cranial nerve ganglia and can reactivate later in life [3–6]. The incidence of HZ increases markedly with age, with risk increasing around 50 years old [7].

A report demonstrated that Rhesus macaques (RM) experimentally infected with simian varicella virus (SVV), the simian counterpart of human VZV, develop primary infection and establish viral latency as occurs in VZV-infected humans. Depletion of CD4+ T cells in these animals resulted in viral reactivation, supporting a pivotal role for CD4+ T-cell immunity in controlling latency [8]. Although CD8+ T cells are considered important for containment of viral infections [9], loss of CD8+ T cells in an SVV-infected RM acute model resulted in slightly higher viral loads and prolonged viremia, while CD4 depletion led to higher viral loads, prolonged viremia, and disseminated varicella [10].

Although most T cells that exhibit cytotoxic function are CD8+ T cells, a subset of differentiated CD4+ T cells also possess cytotoxic ability. This subset is referred to as CD4+ CTLs [11, 12].

CD4+ CTLs have been reported in humans with chronic viral infections including vaccinia, BK virus, and Dengue, among others [9, 11, 13–17]. VZV-specific *in vitro* derived CD4+ T-cell clones have also been shown to exhibit cytolytic activity against VZV-infected B cells [18, 19]. Furthermore, CD4+ CTL responses correlate with clinical outcomes of viral infections, suggesting a significant role in adaptive cellular antiviral immunity [20–22].

Since CD8+ T cells are reported to have limited influence on preventing HZ reactivation [8, 22–28], we proposed that CD4+ T-cell helper function and cytotoxic potential may play a more important role in protecting against VZV reactivation than CD8+ T cells. To address this, we examined the phenotype, functionality, and transcriptomics of CD4+ T cells from people during acute HZ reactivation and people with prior HZ.

## METHODS

### Samples and Individuals

Blood samples were obtained from consented individuals at the Center for Clinical Studies at the University of Texas Health Science Center at Houston, Cleveland VA, or Case Western Reserve University under IRB approved protocols. All samples were collected in sodium heparin tubes and processed at CWRU laboratory after either overnight early arrival shipping (samples from Texas) or overnight storage at room temperature (Cleveland VA and Case Western Reserve University) to more normalize handling from the sites. Peripheral blood mononuclear cells (PBMCs) were isolated from heparinized whole blood by Ficoll gradient centrifugation and cryopreserved in liquid nitrogen until use in assays.

### *In Vitro* Stimulation

PBMC were thawed and rested at 37°C in RPMI-1640 containing 10% FCS for 2 hours then incubated for 2 hours with sucrose gradient purified inactivated VZV (Meridian Life Science, ELLEN strain), costimulatory antibodies anti-CD28 (eBioscience; CD28.2 [1 µg/mL]) and anti-CD49d (BioLegend; 9F10 [1 µg/mL]), and anti-CD107a-FITC (H4A3; BD Biosciences). Staphylococcal enterotoxin B (SEB, Sigma, 1 µg/mL) as a positive control and medium only as a negative control were utilized. After 2 hours, Brefeldin A (Sigma; 5 µg/mL) and monensin (BioLegend; 2µM) were added. After overnight stimulation at 37°C, cells were washed with PBS before staining.

### Flow Cytometry

After stimulation, cells were stained for surface and intracellular markers as performed previously [23, 24]. The following antibodies and dyes were used; Aqua LIVE/DEAD (Invitrogen), anti-CD3 BUV396 (UCHT1; BD Biosciences), anti-CD19 BV510 (SJ25C1; BioLegend), anti-CD14 BV510 (M5E2; BioLegend), anti-CD4 BUV805 (SK3; BD Biosciences), anti-CD8 BV786 (RPA-T8; BD Biosciences), anti-CCR7 PE/Cy7 (G043H7; BioLegend), anti-CD45RA PE/TR (MEM-56; Invitrogen), anti-KLRG1 eFlour710/PerCP (13F12F2; eBioscience), anti-IFN-γ AF700 (B27; BioLegend), anti-IL-2 APC/Cy7 (MQ1-17H12; BioLegend), anti-TNF-α Pacific Blue (MAb11; BioLegend), anti-Perforin-1 PE (B-D48; Cell Sciences), anti-Granzyme B PE/Cy5.5 (GB11; Invitrogen), anti-T-bet BV605 (4B10; BioLegend), and anti-Eomesodermin (EOMES) eFlour660 (WD1928; eBioscience). Flow cytometric analysis was performed on a LSRFortessa (BD Biosciences) and analyzed by Flowjo v10.6.1. Statistics analysis was done by SPICE V6 [25] and GraphPad Prism 8.

### Bulk RNA-Seq and Bioinformatic Analysis

Total memory CD4+ T cells from individuals with acute and prior history of zoster were purified to >99% using live/dead, CD4, CD45RA, and CCR7 markers using a FACSAria flow sorter (BD Biosciences). All individuals with samples still available were run. RNA was purified from memory CD4+ T cells using RNeasy Micro Kits (Qiagen), followed by low input RNASeq library generation using Takara SMART-Seq v4 Ultra Low/Nextera XT with Nextera Index v2 Set A. Paired-end sequencing reactions were run on an Illumina NextSeq 550 High Output platform (30 million total reads per sample). Raw demultiplexed fastq paired-end read files were trimmed off adapters and filtered using the program Skewer to remove reads with an average Phred quality score of less than 30 or trimmed to a length of less than [26]. Trimmed reads were then aligned using the HISAT2 aligner to the *Homo sapiens* NCBI reference genome assembly version GRCh38 and sorted using SAMtools [27, 28]. Aligned reads were counted and assigned to gene meta-features using the program featureCounts as part of the Subread package [29]. These count files were imported into the R programming language and were assessed for quality control, normalized, and analyzed, utilizing the limma-trend method for differential gene expression testing, as well as regression modeling and GSVA using the Bioconductor library [30]. Linear regression modeling was performed using the limma framework [31].

## RESULTS

### Study Cohort

Table 1 describes the demographics of the individuals enrolled; 29 individuals had acute HZ 0 to 12 days prior to enrollment and 16 individuals had prior HZ over 7 months to 45 years prior to enrollment. Individuals with acute zoster had single dermatome zoster and none had disseminated disease. Individuals were adults over age 18, and none were on immunosuppressive medications, had known immunocompromising conditions, or had received prior anti-VZV vaccines. Racial, gender, and age composition are depicted for the 2 groups (Table 1).

**Table 1.** Individual Demographics and Clinical Information

Group		Acute HZ		Prior HZ	
Sex		N	Percentage (%)	N	Percentage (%)
	Male	13	44.8	6	37.5
	Female	16	55.2	10	62.5
Ethnic Group		N	Percentage (%)	N	Percentage (%)
	Asian	2	6.9	3	18.8
	African American	3	10.3	1	6.3
	Caucasian	18	62.1	11	68.8
	Hispanic	6	20.7	1	6.3
Age	Average (range)	49.9±20 (20–82)		42.9±17 (19–71)	
	Median	42		39.5	
Time since HZ	Median (range)	4 days (0–12 days)		5 years (0.54–45 years)	

### Different Cytokine Expression Levels Related to Acute vs Prior HZ

**Figure 1.**
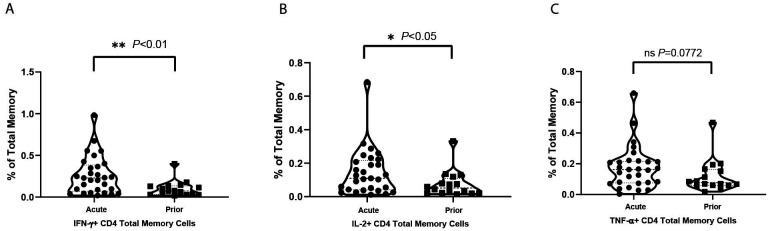
**Violin plots for cytokine positive T-cell percentages under VZV stimulation in total memory CD4+ T cells.** Net positive (VZV stim – no stim control) displayed as % positive with each dot as a unique individual: (A) IFN-γ positive total memory CD4+ T-cell percentages of total memory T cells; (B) IL-2 positive total memory CD4+ T-cell percentages of total memory T cells; (C) TNF-α positive total memory CD4+ T-cell percentages of total memory T cells.

To initially characterize the nature of VZV-specific CD4+ T-cell responses in the prior vs acute reactivation HZ phases, we sampled blood of individuals (n=29) with acute HZ as a result of reactivation of VZV (acute HZ group) and persons with a prior history of HZ (n=16) (prior HZ group). After overnight stimulation of PBMCs with whole inactivated VZV or mitogen, we assessed differences in T-cell memory distribution and VZV-specific CD4+ and CD8+ T-cell responses. Since most donors had very low CD4+ T_EMRA_ cell frequencies, this memory subset was not specifically studied to prevent biased results due to very low cell numbers for analysis. CD4+ total memory cells had readily detectable VZV-specific cells in most individuals (Figure 1). In contrast, we detected very low VZV-specific CD8+ T-cell responses (median below limit of detection <0.01% in TNF-α, IL-2, IFN-γ) (Supplementary Figure 1). Due to these very limited responses compared to the much more robust CD4+ responses, we focused our analysis on CD4+ T cells only.

The frequencies of total memory CD4+ T cells (Figure 1), central memory (CM) (Supplementary Figure 2A) and effector memory (EM) (Supplementary Figure 2B) subsets responding to VZV stimulation are shown. Over twice as many of the cytokine-producing CD4+ T cells induced by VZV were EM cells with the frequency of CM cells producing cytokines being notably lower. Within the EM population, TNF-α+, IL-2+, and IFN-γ+ cells were significantly (*P*<0.05, *P*<0.05, and *P*<0.01, respectively) more frequent in the acute HZ individuals than prior HZ individuals. These results suggest that viral control is mostly carried out by EM T cells during the acute phase of HZ. Meanwhile, IL-2+ cells were also significantly (*P*<0.05) more frequent in the CM population of acute HZ individuals. Similar trends in IL-2 and IFN-γ are also present in total memory T cells. After mitogen staphylococcal enterotoxin B (SEB) stimulation, not surprisingly, there are no significant differences in the frequencies of IL-2, IFN-γ, or TNF-α-producing cells elicited between the acute HZ and prior HZ in CM, EM, or total memory CD4+ T cells.

Similar to Weinberg et al [32], we found a correlation between time that rash has been present and magnitude of VZV-specific IFN-γ secreting cells (*r*=0.3831, *P*=0.0646) (Supplementary Figure 3A) and a trend with CD107a (*r*=0.6042, *P*=0.0018) (Supplementary Figure 3B). This suggests that cellular immunity against VZV is gradually reduced after the end of the acute HZ phase.

### Different Polyfunctional Patterns Between Individuals with Acute HZ and Prior HZ History

**Figure 2.**
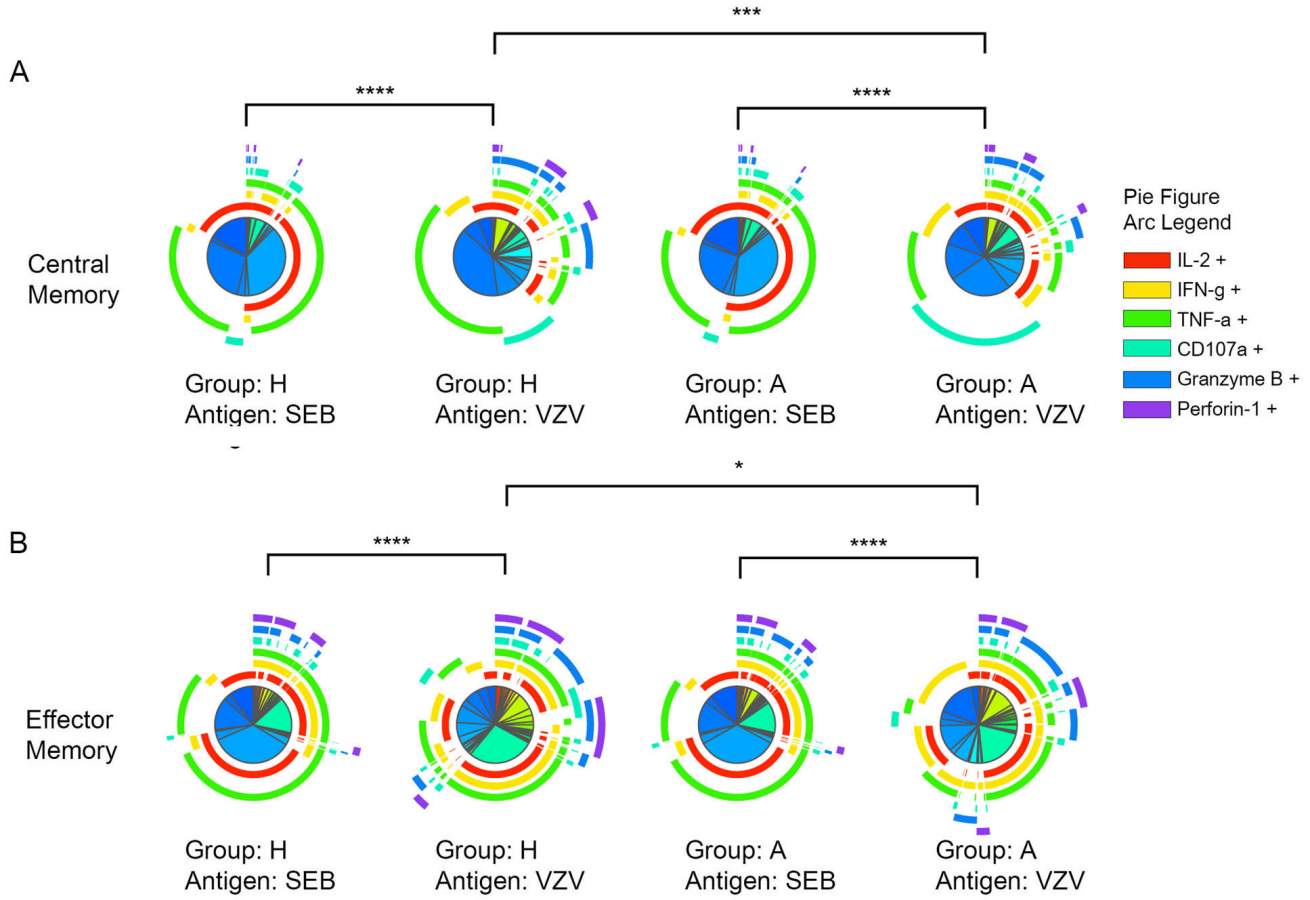
**Significant polyfunction differences of central and effector memory CD4+ T cells elicited by SEB or VZV in different groups of individuals.** The pie charts represent different combinations of cytokine and effector molecule expression patterns of (A) central memory CD4+ T cells; (B) effector memory CD4+ T cells. The arcs are color coded for each effector function with the color legend noted. (*:*P*<0.05; **:*P*<0.01; ***:*P*<0.001) against SEB or VZV in acute HZ (A) and prior HZ (H) groups.

After VZV stimulation, CD4+ CM as well as CD4+ EM T-cell subsets varied significantly in polyfunctional response patterns between acute HZ and prior HZ group which indicates a different VZV-specific CD4+ T-cell response depending on the presence of acute HZ (Figure 2A and B). Also, CM and EM CD4+ T cells are more polyfunctional responding to VZV than the mitogen, SEB. The polyfunction elicited by SEB had no difference between acute HZ and prior HZ groups, which suggests that polyfunction differences are VZV-specific and not individual group specific.

### Cytotoxicity Phenotype of VZV-Specific CD4+ T Cells

**Figure 3.**
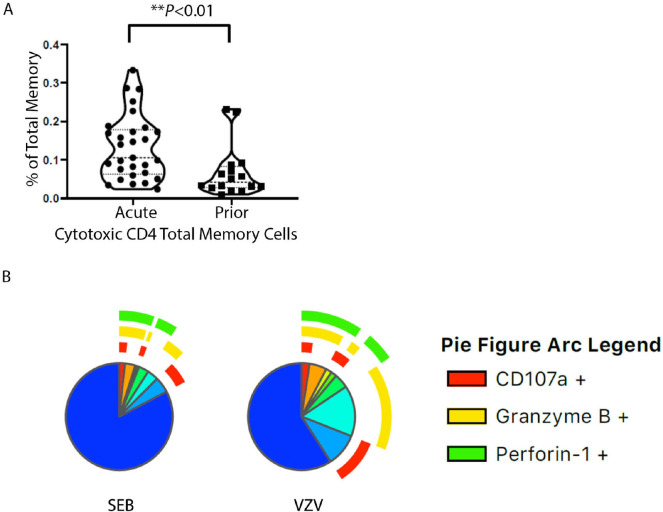
(A) Violin plots for cytotoxic CD4+ T-cell percentages in total memory CD4+ T cells responding to VZV stimulation cytotoxic CD4+ T-cell percentage of total memory CD4+ T cells. Each dot represents a unique individual. (B) Pie charts showing the proportion and expression pattern of CD4+T-cell cytotoxic function after SEB or VZV stimulation in all individuals. Blue region without arcs depicts non-cytotoxic functions.

PBMCs were stimulated with VZV-antigen in order to identify which cells are VZV-specific. Considering the constitutive expression of Perforin-1 and Granzyme B in T cells, T cells that had VZV-induced IL-2, IFN-γ, or TNF-α or exocytosis marker CD107a were then analyzed for co-expression of Perforin-1 and or Granzyme B. Cells in this group that expressed any one or more of the CTL molecules including CD107a, Perforin-1, and Granzyme B were hereby classified as VZV-specific T cells with expression of cytotoxic molecules. The frequency of those cytotoxic CD4+ T cells is higher in total memory CD4+ T cells in acute compared to prior HZ, with the EM subset accounting for that difference (Figure 3A and Supplementary Figure 4A and B). The proportion of cells with expression of cytotoxic molecules is greater in EM than CM as anticipated. When grouping the VZV or mitogen SEB responding cells and assessing cytotoxic molecule expression of only the responding cells, there is significantly more cytotoxic molecule expression in the VZV responding than mitogen (*P*<0.021) (Figure 3B). The brefeldin and monensin block transport of newly formed molecules, but pre-formed Perforin-1 and Granzyme B may still be released upon stimulation meaning these results may even be an undercount of cells with cytotoxic molecule expression.

This suggests CD4+ T cells initially exposed to VZV that became memory VZV-specific CD4+ T-cell subsets have greater cytotoxic molecule expression than the non-VZV-specific memory cells that we examined with SEB as the non-specific mitogenic stimuli. It suggests that the immune context of VZV infection at the time of differentiation and/or expansion favors the cytotoxic effector function of the VZV-specific CD4+ T cells.

### Higher T-bet in VZV-Specific T Cells vs Total Memory Cells

**Figure 4.**
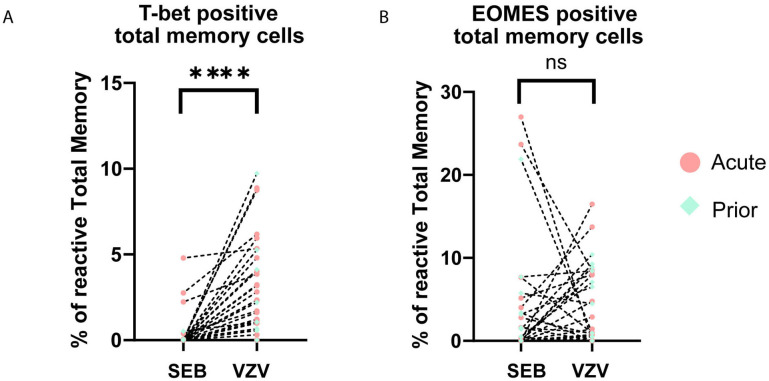
**Dot plots showing the expression of transcription factors.** (A) T-bet and (B) EOMES in total memory CD4+ T cells upon stimulation by VZV and SEB in all samples. Each dot represents a unique individual. Dotted line connected data points are from the same individual. (red dot: acute HZ group; green dot: prior HZ group)

During differentiation, T-bet and EOMES expressions are essential for certain effector functions [33–36]. There was no significant difference in the frequency of T-bet or EOMES expressing cells in VZV-responding cells between acute HZ and prior HZ groups (CM:*P*=0.07; EM:*P*=0.10; Total memory: 0.30) (Supplementary Figure 5). However, when combining all the patients’ data to reach a larger sample size, we found T-bet expression of memory CD4+ T cells to be higher in reactive VZV-reactive compared to SEB-reactive total memory for all patients (*P*<0.0001) (Figure 4A), but EOMES does not have this pattern (Figure 4B). Meanwhile, the difference in T-bet level also exists in reactive CM and EM subsets (Supplementary Figure 6), which indicates the sub-set-independent upregulation in T-bet for VZV-specific T cells. These sets of data suggest a different pattern of T-bet expression in VZV-specific cells regardless of whether the individual had acute HZ or a prior HZ. T-bet is essential for certain effector functions like IFN-γ expression and Th1 polarization. Differential T-bet expression may be the reason for differences in T-cell effector function and polyfunctionality that we have seen in VZV-specific cells.

### Identification of Objectively Determined Differences in Coordinate Protein Expression Levels in Total CD4+ T Cells by Uniform Manifold Approximation and Projection (UMAP)

**Figure 5.**
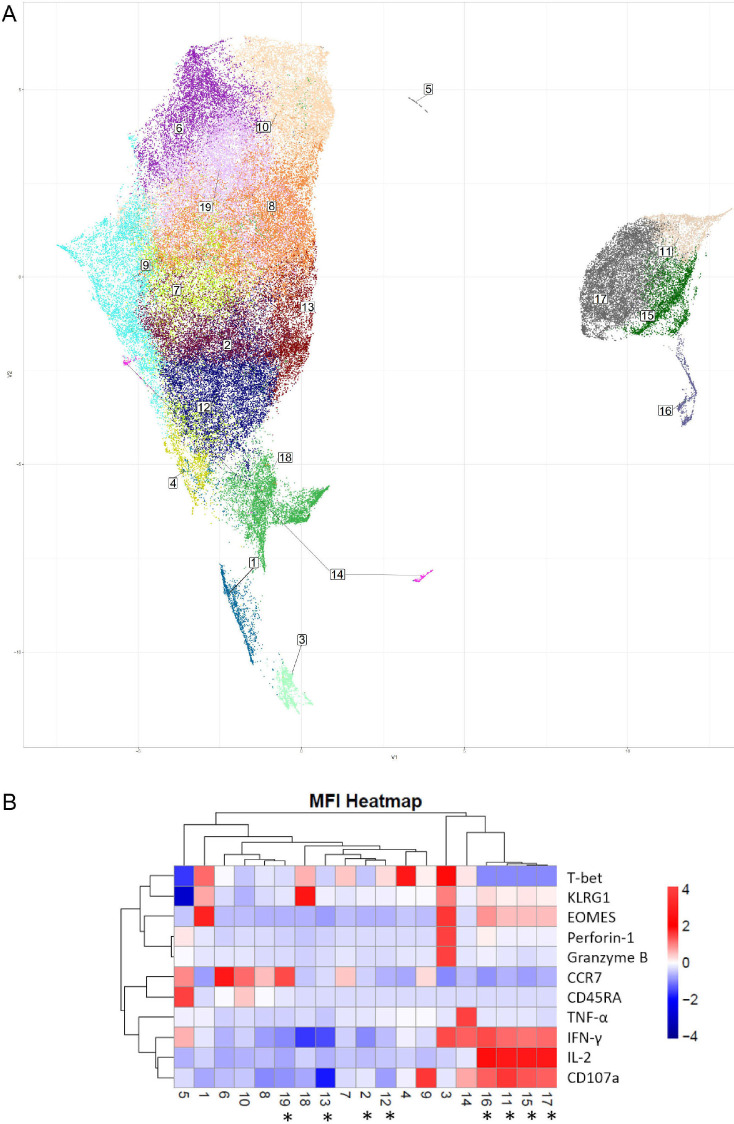
**Dimension reduction (Uniform Manifold Approximation and Projection or UMAP) analysis of phenotypic flow cytometric data from acute and prior group samples**. (A) UMAP analysis of total CD4+ T-cell response upon VZV stimulation with cluster numbers indicated, (B) heatmap showing the normalized expression levels of T-cell markers and effector molecules for each indicated cluster based on MFI. Asterisks denote statistically significant differences in cluster frequencies between the acute and prior groups (*:*P*<0.05).

To provide deeper insight into differences in coordinate protein expression during acute HZ reactivation vs prior HZ history, unsupervised dimension reduction via UMAP was used for further analysis of the total CD4+ T-cell flow cytometric data on samples from VZV individuals. UMAP analysis provides a highly reproducible and objective representation of dimensionality of multiparametric data by clustering cells with similar phenotypes [37–39]. UMAP identified several groups that have significant differences in frequency between individuals in acute HZ and those with prior HZ (Figure 5A). Clusters 2, 11, 12, 13, 15, 17, and 19 are significantly enriched in individuals with acute vs prior HZ (Figure 5B). Clusters 11, 15, and 17 which demonstrated significantly differential coordinate expression, including significant expansion in acute HZ individuals, were almost absent in prior HZ individuals. In Figure 5B, a heatmap displaying normalized expression of the proteins by mean fluorescent intensity for each of the UMAP identified clusters shows that they likely belong to a group in the EM compartment by their relatively lower CCR7 and lower CD45RA expression levels, and they also exhibit high levels of EOMES, IL-2 and IFN-γ expression. Meanwhile, their high CD107a levels suggest that that may have been more likely to have degranulated upon re-stimulation.

Although not significantly enriched in the acute HZ group, cluster 3 shows intermediate Perforin-1 and Granzyme B levels, which correspond with cytotoxic CD4+ T-cell function. It suggests the role of cytotoxic CD4+ T cells in anti-VZV immunity based on their close clustering with other antigen-specific CD4+ T cells. Many of those antigen-specific CD4+ T-cell subsets are significantly more abundant in people during the acute phase of HZ and are EOMES expressing, while there is only a limited T-bet expression in those clusters. It may suggest CD4+ effector functions are mostly driven by EOMES in this case [34, 40, 41]. In other words, EOMES loss in VZV-specific cells may be associated with VZV reactivation.

**Figure 6.**
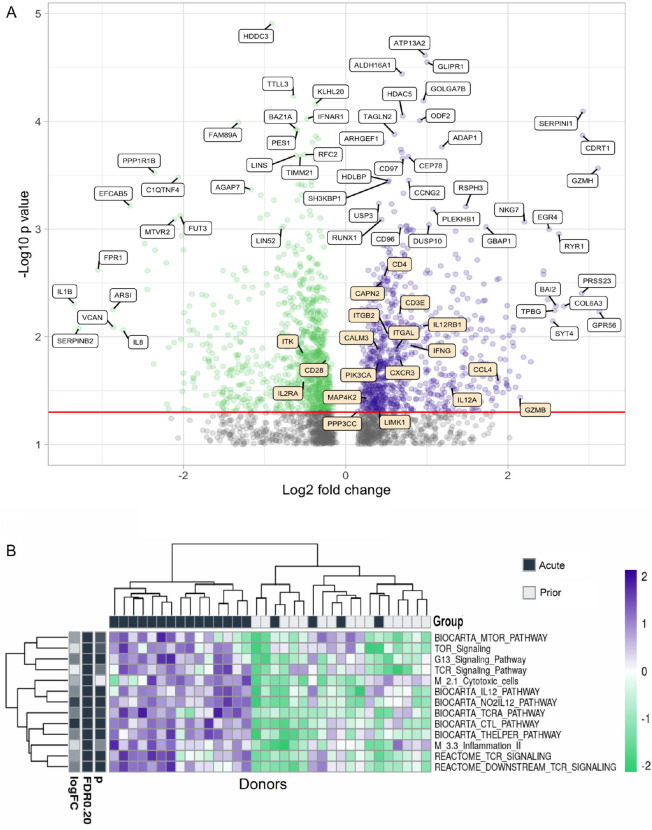
**Gene expression and pathway analysis of CD4+ memory T cells sorted from PBMCs from acute and prior individuals.** (A) Volcano plot showing the differences in gene expression in memory CD4+ T cells in acute group donors vs prior group donors. Most significantly upregulated genes by *P* value and by log2-fold change are indicated with white labels, while genes from the BIOCARTA T-cell pathway are indicated in orange. This pathway includes the following: *‘BIOCARTA_CTL_PATHWAY’, ‘BIOCARTA_CTLA4_PATHWAY’, ‘BIOCARTA_MAL_PATHWAY’, ‘BIOCARTA_MEF2D_PATHWAY’, ‘BIOCARTA_NKT_PATHWAY’, and ‘BIOCARTA_TOB1_PATHWAY’.* (B) Heatmap of different pathway activation patterns of CD4+ T cells from donors in acute group and prior group. Donors are clustered by pathway patterns. (*:*P*<0.05).

RNAseq of the flow cytometry purified total CD4+ memory T cells was also performed. Several genes have significantly different (*P*<0.05) expression levels between people with acute HZ or prior HZ history (Figure 6A). Some genes related to T-cell function such as ITGAM and Granzyme H are more highly expressed during the acute reactivation phase, while IL-1b, IL-8, C1QTNF4, and IFNAR1 are more expressed in individuals with prior HZ. Those observations suggest more CD4+ effector function during the acute phase while more chronic inflammation occurs afterwards.

In the pathway enrichment analysis, it is clear that TCR activation, mTOR, IL-12, CTL, T helper, and inflammation pathways have significant differences between people with different VZV infection history (Figure 6B). These pathways are more enriched in individuals during acute HZ reactivation. Also, the activation patterns of these pathways co-cluster with the prior HZ status of these individuals, which suggests a high level of T-cell effector functions including cytokine release and cytotoxicity. In addition, it shows the active differentiation and metabolism of the T cells during acute HZ, including differential expression of TCR, CTL, T helper, inflammation, and MTOR signaling (*P*<0.05 for genes/pathways).

## DISCUSSION

A full understanding of cellular immunity against VZV infection remains unclear. CD8+ T cells show limited function in viral clearance based on the primate SVV models; for this reason we focused our experiments and subsequent analysis on methods that would optimally elicit CD4+ T cells that included use of the whole VZV virus as the stimulating antigen rather than just peptides. In this study, we used multicolor flow cytometry and RNA-seq to elucidate the mechanisms of control of VZV reactivation between acute HZ and prior history of HZ. Our major findings include: (1) distinct cytokine expression levels in IFN-γ and IL-2 between groups of individuals with acute vs prior HZ, which are mostly accounted for by CD4+ EM T cells; (2) a distinct VZV-specific cytotoxic CD4+ T-cell population that was also different in persons with acute vs prior HZ; (3) CD4+ T cells from people with acute vs prior HZ showed distinct polyfunctional patterns; (4) UMAP analysis of clustering total CD4+ T cells showed 19 distinct T-cell clusters among which 7 clusters have significant differences between people with acute vs prior HZ; and (5) multiple differences in differentiation and inflammatory genes in memory CD4+ T cells from acute and prior HZ individuals were determined by RNA-seq analysis.

We propose the role of CD4+ T cells during HZ includes not only the classical T-helper functions eg, IL-2, IFN-γ, and TNF-α secretion to orchestrate cellular and humoral immunity, but also a non-classical Perforin-1 and Granzyme B-mediated cytotoxic function. Such CD4+ cytotoxic function is also found in some other viral infections [12–15, 17]. Cytotoxic CD4+ T-cell characteristics are primarily found by EM cells rather than CM cells. The identification of differences in cytotoxic CD4+ EM T cells between people with different phases of VZV infection status suggests the cytotoxic CD4+ EM T-cell population is shrinking over time after initial acute VZV infection. This may account for the loss of cytotoxic cellular immunity and lead to the reactivation of VZV. Compared to the loss of VZV-specific effector function in CM T cells, the EM T cells seem to be more vulnerable to effector function loss over time which suggests that the number of VZV-specific CD4+ EM cells decreases, and the control of VZV infection is diminished with time.

Previous studies generated *in vitro* VZV-specific CD4+ T-cell clones that could lyse target cells [18, 19]. Our studies described the CTL molecules *ex vivo* and showed heightened CTL molecules in VZV-specific cells compared to the general population of memory T cells supporting unique differences. Moreover, research in other viral infections have shown CD4+ T cells with cytotoxic phenotype in Dengue Virus and SARS-CoV-2 [16, 42]. Interestingly, those cytotoxic CD4+ T cells express chemokine receptors like CX3CR1, CCL3, CCL4, and CCL5, which suggests recruitment to the infection site [13, 15–17, 42], and in Meckiff et al [42], the SARS-CoV-2-reactive CD4+ CTLs showed similar UMAP clustering patterns as our study.

Schub et al compared acute HZ to a healthy control population that included a range of ages, prior history of VZV vaccine, prior chickenpox, or HZ. Similar to our findings, acute HZ individuals had higher responses than controls, but different from our findings, their controls had a higher polyfunctional proportion of IL-2/TNF-α/IFN-γ cells than acute HZ. The differences could be due to all our comparator control individuals having a prior history of HZ and/or our acute individuals being tested earlier in the acute phase than in their study [43]. They also found that 4 months after acute HZ, on resampling their responses reverted to the same as their control group that never had HZ. Weinberg et al also described this similar phenomenon in humoral and cellular immunity, after VZV activation, ie, the VZV-specific IFN-γ expressing T cells and VZV-specific antibody titer rise with time, peak at 3 weeks, then decrease over time [32]. This agrees with our hypothesis that after the active phase, cellular immunity to VZV will be reduced or lost over time.

Aging is also another factor to consider during VZV reactivation. In our study, there was a modest (*r*= -0.331) but significant (*P*<0.05) negative correlation between age and VZV-specific IFN-γ percentage of CD4+ total memory T cells, which suggests aging leads to loss of antigen-specific cellular immunity and may allow reactivation. Several studies in the literature assessing T-cell responses to VZV in persons with childhood VZV infection or vaccine demonstrated diminished response with age [44]. They also speculated that may contribute to increased reaction with aging.

RNA-seq of purified memory CD4+ T cells revealed different T-cell activation, inflammation, and metabolism pathways including IFN, TNF, and MTOR signaling in CD4+ memory T cells from individuals that had acute vs. prior HZ. In addition, ITGAM (CD11b) is significantly more expressed (*P*<0.05 for genes/pathways) in acute HZ individuals. CD11b is a marker for CD4+ T-cell activation, proliferation, and cytotoxicity. Granzyme H, a cytotoxic effector molecule, is also more expressed in acute HZ individuals suggesting CD4+ T cells may have more cytotoxic potential during active VZV reactivation, which is consistent with the UMAP and flow cytometry results. IL-1β, IL-8, C1QTNF4, and IFNAR1 are less expressed in acute individuals. IL-1β is an important mediator of inflammatory response, and also engaged in multiple immune processes. IL-8 is related to neutrophil chemotaxis. C1QTNF4 shows context dependent pro- or anti-inflammatory effects. IFNAR1 encodes the type I interferon receptor. Upon binding with the ligand, it would trigger the activation of JNKs and the downstream signals.

T cells showed stronger activation pathways and more active metabolism during the HZ acute phase. These findings correspond with previous results from primate SVV models [45]. In addition, an SVV primate model study focused on subclinical reactivation and found that viral reactivation leads to greater changes in ganglia gene expression than latent viral infection mostly in cell cycle regulation, metabolism, and stress response [45].

Given the fact that CD8+ T cells show limited engagement in cellular immunity against VZV in animal models [10], and the discovery of a cytotoxic CD4+ T cell population in active HZ, cytotoxic CD4+ T cells may be responsible for MHC-II restricted killing of the viral-infected cells [11]. Further studies around this cell population could be done to help understand the cellular immunity against VZV and serve as a basis for future design of VZV and other herpes virus vaccines.

## SUPPLEMENTARY DATA

Supplementary materials are available at the *Pathogens and Immunity* website. Supplementary data may be provided by the authors to benefit the reader. Supplementary data are not copyedited and are the sole responsibility of the authors. Questions or comments related to supplementary materials should be addressed to the corresponding author.

## References

[1] Davison AJ, Scott JE. The complete DNA sequence of varicella-zoster virus. J Gen Virol. 1986;67 (Pt 9):1759–816. Epub 1986/09/01. doi: 10.1099/0022-1317-67-9-1759. PubMed PMID: 3018124. 3018124

[2] John AR, Canaday DH. Herpes Zoster in the Older Adult. Infect Dis Clin North Am. 2017;31(4):811–26. Epub 2017/10/29. doi: 10.1016/j.idc.2017.07.016. PubMed PMID: 29079160; PMCID: PMC5724974. 29079160 PMC5724974

[3] Gilden DH, Cohrs RJ, Mahalingam R. Clinical and molecular pathogenesis of varicella virus infection. Viral Immunol. 2003;16(3):243–58. Epub 2003/10/30. doi: 10.1089/088282403322396073. PubMed PMID: 14583142. 14583142

[4] Kennedy PGE. Neurological complications of varicella-zoster virus infections. : Butterworth; 1987.

[5] Johnson RT. Viral infections of the nervous system. Philadelphia: Lippincott-Raven Publishers; 1998.

[6] Gershon AA, Breuer J, Cohen JI, Cohrs RJ, Gershon MD, Gilden D, Grose C, Hambleton S, Kennedy PG, Oxman MN, Seward JF, Yamanishi K. Varicella zoster virus infection. Nat Rev Dis Primers. 2015;1:15016. Epub 20150702. doi: 10.1038/nrdp.2015.16. PubMed PMID: 27188665; PMCID: PMC5381807. 27188665 PMC5381807

[7] Kawai K, Yawn BP, Wollan P, Harpaz R. Increasing Incidence of Herpes Zoster Over a 60-year Period From a Population-based Study. Clin Infect Dis. 2016;63(2):221–6. Epub 20160508. doi: 10.1093/cid/ciw296. PubMed PMID: 27161774; PMCID: PMC4928389. 27161774 PMC4928389

[8] Traina-Dorge V, Palmer BE, Coleman C, Hunter M, Frieman A, Gilmore A, Altrock K, Doyle-Meyers L, Nagel MA, Mahalingam R. Reactivation of Simian Varicella Virus in Rhesus Macaques aﬅer CD4 T Cell Depletion. J Virol. 2019;93(3). Epub 20190117. doi: 10.1128/JVI.01375-18. PubMed PMID: 30404798; PMCID: PMC6340024. PMC6340024 30404798

[9] Binder D, Kundig TM. Antiviral protection by CD8+ versus CD4+ T cells. CD8+ T cells correlating with cytotoxic activity in vitro are more efficient in antivaccinia virus protection than CD4-dependent IL. J Immunol. 1991;146(12):4301–7. Epub 1991/06/15. PubMed PMID: 1710246. 1710246

[10] Haberthur K, Engelmann F, Park B, Barron A, Legasse A, Dewane J, Fischer M, Kerns A, Brown M, Messaoudi I. CD4 T cell immunity is critical for the control of simian varicella virus infection in a nonhuman primate model of VZV infection. PLoS Pathog. 2011;7(11):e1002367. Epub 20111110. doi: 10.1371/journal.ppat.1002367. PubMed PMID: 22102814; PMCID: PMC3213099. 22102814 PMC3213099

[11] Juno JA, van Bockel D, Kent SJ, Kelleher AD, Zaunders JJ, Munier CM. Cytotoxic CD4 T Cells-Friend or Foe during Viral Infection? Front Immunol. 2017;8:19. Epub 20170123. doi: 10.3389/fimmu.2017.00019. PubMed PMID: 28167943; PMCID: PMC5253382. 28167943 PMC5253382

[12] Takeuchi A, Saito T. CD4 CTL, a Cytotoxic Subset of CD4(+) T Cells, Their Differentiation and Function. Front Immunol. 2017;8:194. Epub 20170223. doi: 10.3389/fimmu.2017.00194. PubMed PMID: 28280496; PMCID: PMC5321676. 28280496 PMC5321676

[13] Kurane I, Brinton MA, Samson AL, Ennis FA. Dengue virus-specific, human CD4+ CD8-cytotoxic T-cell clones: multiple patterns of virus cross-reactivity recognized by NS3-specific T-cell clones. J Virol. 1991;65(4):1823–8. Epub 1991/04/01. doi: 10.1128/JVI.65.4.1823-1828.1991. PubMed PMID: 1705990; PMCID: PMC239991. 1705990 PMC239991

[14] Zhou W, Sharma M, Martinez J, Srivastava T, Diamond DJ, Knowles W, Lacey SF. Functional characterization of BK virus-specific CD4+ T cells with cytotoxic potential in seropositive adults. Viral Immunol. 2007;20(3):379–88. Epub 2007/10/13. doi: 10.1089/vim.2007.0030. PubMed PMID: 17931108. 17931108

[15] Tian Y, Sette A, Weiskopf D. Cytotoxic CD4 T Cells: Differentiation, Function, and Application to Dengue Virus Infection. Front Immunol. 2016;7:531. Epub 20161207. doi: 10.3389/fimmu.2016.00531. PubMed PMID: 28003809; PMCID: PMC5141332. 28003809 PMC5141332

[16] Weiskopf D, Bangs DJ, Sidney J, Kolla RV, De Silva AD, de Silva AM, Crotty S, Peters B, Sette A. Dengue virus infection elicits highly polarized CX3CR1+ cytotoxic CD4+ T cells associated with protective immunity. Proc Natl Acad Sci U S A. 2015;112(31):E4256-63. Epub 20150720. doi: 10.1073/pnas.1505956112. PubMed PMID: 26195744; PMCID: PMC4534238. 26195744 PMC4534238

[17] Kurane I, Zeng L, Brinton MA, Ennis FA. Definition of an epitope on NS3 recognized by human CD4+ cytotoxic T lymphocyte clones cross-reactive for dengue virus types 2, 3, and 4. Virology. 1998;240(2):169–74. Epub 1998/02/10. doi: 10.1006/viro.1997.8925. PubMed PMID: 9454689. 9454689

[18] Huang Z, Vafai A, Lee J, Mahalingam R, Hayward AR. Specific lysis of targets expressing varicella-zoster virus gpI or gpIV by CD4+ human T-cell clones. J Virol. 1992;66(5):2664–9. Epub 1992/05/01. doi: 10.1128/JVI.66.5.2664-2669.1992. PubMed PMID: 1348545; PMCID: PMC241020. 1348545 PMC241020

[19] Hayward AR, Pontesilli O, Herberger M, Laszlo M, Levin M. Specific lysis of varicella zoster virus-infected B lymphoblasts by human T cells. J Virol. 1986;58(1):179–84. Epub 1986/04/01. doi: 10.1128/JVI.58.1.179-184.1986. PubMed PMID: 3005647; PMCID: PMC252891. 3005647 PMC252891

[20] Marshall NB, Swain SL. Cytotoxic CD4 T cells in antiviral immunity. J Biomed Biotechnol. 2011;2011:954602. Epub 20111122. doi: 10.1155/2011/954602. PubMed PMID: 22174559; PMCID: PMC3228492. 22174559 PMC3228492

[21] Cheroutre H, Husain MM. CD4 CTL: living up to the challenge. Semin Immunol. 2013;25(4):273–81. Epub 20131115. doi: 10.1016/j.smim.2013.10.022. PubMed PMID: 24246226; PMCID: PMC3886800. 24246226 PMC3886800

[22] Brown DM, Dilzer AM, Meents DL, Swain SL. CD4 T cell-mediated protection from lethal influenza: perforin and antibody-mediated mechanisms give a one-two punch. J Immunol. 2006;177(5):2888–98. Epub 2006/08/22. doi: 10.4049/jimmunol.177.5.2888. PubMed PMID: 16920924. 16920924

[23] Canaday DH, Wilkinson RJ, Li Q, Harding CV, Silver RF, Boom WH. CD4(+) and CD8(+) T cells kill intracellular Mycobacterium tuberculosis by a perforin and Fas/Fas ligand-independent mechanism. J Immunol. 2001;167(5):2734–42. Epub 2001/08/18. doi: 10.4049/jimmunol.167.5.2734. PubMed PMID: 11509617. 11509617

[24] Van Epps P, Banks R, Aung H, Betts MR, Canaday DH. Age-related differences in polyfunctional T cell responses. Immun Ageing. 2014;11(1):14. Epub 20141023. doi: 10.1186/1742-4933-11-14. PubMed PMID: 25512758; PMCID: PMC4265991. 25512758 PMC4265991

[25] Roederer M, Nozzi JL, Nason MC. SPICE: exploration and analysis of post-cytometric complex multivariate datasets. Cytometry A. 2011;79(2):167–74. Epub 20110107. doi: 10.1002/cyto.a.21015. PubMed PMID: 21265010; PMCID: PMC3072288. 21265010 PMC3072288

[26] Jiang H, Lei R, Ding SW, Zhu S. Skewer: a fast and accurate adapter trimmer for next-generation sequencing paired-end reads. BMC Bioinformatics. 2014;15:182. Epub 20140612. doi: 10.1186/1471-2105-15-182. PubMed PMID: 24925680; PMCID: PMC4074385. 24925680 PMC4074385

[27] Kim D, Paggi JM, Park C, Bennett C, Salzberg SL. Graph-based genome alignment and genotyping with HISAT2 and HISAT-genotype. Nat Biotechnol. 2019;37(8):907–15. Epub 20190802. doi: 10.1038/s41587-019-0201-4. PubMed PMID: 31375807; PMCID: PMC7605509. 31375807 PMC7605509

[28] Li H, Handsaker B, Wysoker A, Fennell T, Ruan J, Homer N, Marth G, Abecasis G, Durbin R, Genome Project Data Processing S. The Sequence Alignment/Map format and SAMtools. Bioinformatics. 2009;25(16):2078–9. Epub 20090608. doi: 10.1093/bioinformatics/btp352. PubMed PMID: 19505943; PMCID: PMC2723002. 19505943 PMC2723002

[29] Liao Y, Smyth GK, Shi W. featureCounts: an efficient general purpose program for assigning sequence reads to genomic features. Bioinformatics. 2014;30(7):923–30. Epub 20131113. doi: 10.1093/bioinformatics/btt656. PubMed PMID: 24227677. 24227677

[30] Hanzelmann S, Castelo R, Guinney J. GSVA: gene set variation analysis for microarray and RNA-seq data. BMC Bioinformatics. 2013;14:7. Epub 20130116. doi: 10.1186/1471-2105-14-7. PubMed PMID: 23323831; PMCID: PMC3618321. 23323831 PMC3618321

[31] Ritchie ME, Phipson B, Wu D, Hu Y, Law CW, Shi W, Smyth GK. limma powers differential expression analyses for RNA-sequencing and microarray studies. Nucleic Acids Res. 2015;43(7):e47. Epub 20150120. doi: 10.1093/nar/gkv007. PubMed PMID: 25605792; PMCID: PMC4402510. 25605792 PMC4402510

[32] Weinberg A, Canniff J, Rouphael N, Mehta A, Mulligan M, Whitaker JA, Levin MJ. Varicella-Zoster Virus-Specific Cellular Immune Responses to the Live Attenuated Zoster Vaccine in Young and Older Adults. J Immunol. 2017;199(2):604–12. Epub 20170612. doi: 10.4049/jimmunol.1700290. PubMed PMID: 28607114; PMCID: PMC5505810. 28607114 PMC5505810

[33] Li G, Yang Q, Zhu Y, Wang HR, Chen X, Zhang X, Lu B. T-Bet and Eomes Regulate the Balance between the Effector/Central Memory T Cells versus Memory Stem Like T Cells. PLoS One. 2013;8(6):e67401. Epub 20130627. doi: 10.1371/journal.pone.0067401. PubMed PMID: 23826287; PMCID: PMC3694876. 23826287 PMC3694876

[34] Qui HZ, Hagymasi AT, Bandyopadhyay S, St Rose MC, Ramanarasimhaiah R, Menoret A, Mittler RS, Gordon SM, Reiner SL, Vella AT, Adler AJ. CD134 plus CD137 dual costimulation induces Eomesodermin in CD4 T cells to program cytotoxic Th1 differentiation. J Immunol. 2011;187(7):3555–64. Epub 20110831. doi: 10.4049/jimmunol.1101244. PubMed PMID: 21880986; PMCID: PMC3178659. 21880986 PMC3178659

[35] Lord GM, Rao RM, Choe H, Sullivan BM, Lichtman AH, Luscinskas FW, Glimcher LH. T-bet is required for optimal proinflammatory CD4+ T-cell trafficking. Blood. 2005;106(10):3432–9. Epub 20050712. doi: 10.1182/blood-2005-04-1393. PubMed PMID: 16014561; PMCID: PMC1895048. 16014561 PMC1895048

[36] Mazzoni A, Maggi L, Siracusa F, Ramazzotti M, Rossi MC, Santarlasci V, Montaini G, Capone M, Rossettini B, De Palma R, Kruglov A, Chang HD, Cimaz R, Maggi E, Romagnani S, Liotta F, Cosmi L, Annunziato F. Eomes controls the development of Th17-derived (non-classic) Th1 cells during chronic inflammation. Eur J Immunol. 2019;49(1):79–95. Epub 20181122. doi: 10.1002/eji.201847677. PubMed PMID: 30144030. 30144030

[37] Becht E, McInnes L, Healy J, Dutertre CA, Kwok IWH, Ng LG, Ginhoux F, Newell EW. Dimensionality reduction for visualizing single-cell data using UMAP. Nat Biotechnol. 2018. Epub 20181203. doi: 10.1038/nbt.4314. PubMed PMID: 30531897. 30531897

[38] Lin L, Finak G, Ushey K, Seshadri C, Hawn TR, Frahm N, Scriba TJ, Mahomed H, Hanekom W, Bart PA, Pantaleo G, Tomaras GD, Rerks-Ngarm S, Kaewkungwal J, Nitayaphan S, Pitisuttithum P, Michael NL, Kim JH, Robb ML, O’Connell RJ, Karasavvas N, Gilbert P, S CDR, McElrath MJ, Gottardo R. COMPASS identifies T-cell subsets correlated with clinical outcomes. Nat Biotechnol. 2015;33(6):610–6. Epub 20150525. doi: 10.1038/nbt.3187. PubMed PMID: 26006008; PMCID: PMC4569006. 26006008 PMC4569006

[39] McInnes L HJ, Melville J. Umap: Uniform manifold approximation and projection for dimension reduction. arXiv:180203426. 2018. Epub February 9.

[40] Curran MA, Geiger TL, Montalvo W, Kim M, Reiner SL, Al-Shamkhani A, Sun JC, Allison JP. Systemic 4-1BB activation induces a novel T cell phenotype driven by high expression of Eomesodermin. J Exp Med. 2013;210(4):743–55. Epub 20130401. doi: 10.1084/jem.20121190. PubMed PMID: 23547098; PMCID: PMC3620352. 23547098 PMC3620352

[41] Hirschhorn-Cymerman D, Budhu S, Kitano S, Liu C, Zhao F, Zhong H, Lesokhin AM, Avogadri-Connors F, Yuan J, Li Y, Houghton AN, Merghoub T, Wolchok JD. Induction of tumoricidal function in CD4+ T cells is associated with concomitant memory and terminally differentiated phenotype. J Exp Med. 2012;209(11):2113–26. Epub 20120924. doi: 10.1084/jem.20120532. PubMed PMID: 23008334; PMCID: PMC3478933. 23008334 PMC3478933

[42] Meckiff BJ, Ramirez-Suastegui C, Fajardo V, Chee SJ, Kusnadi A, Simon H, Grifoni A, Pelosi E, Weiskopf D, Sette A, Ay F, Seumois G, Ottensmeier CH, Vijayanand P. Single-cell transcriptomic analysis of SARS-CoV-2 reactive CD4 (+) T cells. bioRxiv. 2020. Epub 20200613. doi: 10.1101/2020.06.12.148916. PubMed PMID: 32587963; PMCID: PMC7310619. PMC7534589 33096020

[43] Schub D, Janssen E, Leyking S, Sester U, Assmann G, Hennes P, Smola S, Vogt T, Rohrer T, Sester M, Schmidt T. Altered phenotype and functionality of varicella zoster virus-specific cellular immunity in individuals with active infection. J Infect Dis. 2015;211(4):600–12. Epub 20140901. doi: 10.1093/infdis/jiu500. PubMed PMID: 25180236. 25180236

[44] Weinberg A, Zhang JH, Oxman MN, Johnson GR, Hayward AR, Caulfield MJ, Irwin MR, Clair J, Smith JG, Stanley H, Marchese RD, Harbecke R, Williams HM, Chan IS, Arbeit RD, Gershon AA, Schodel F, Morrison VA, Kauffman CA, Straus SE, Schmader KE, Davis LE, Levin MJ, Investigators USDoVACSPSPS. Varicella-zoster virus-spe-cific immune responses to herpes zoster in elderly participants in a trial of a clinically effective zoster vaccine. J Infect Dis. 2009;200(7):1068–77. Epub 2009/08/29. doi: 10.1086/605611. PubMed PMID: 19712037; PMCID: PMC4014851. 19712037 PMC4014851

[45] Arnold N, Messaoudi I. Simian varicella virus causes robust transcriptional changes in T cells that support viral replication. Virus Res. 2017;238:226–35. Epub 20170708. doi: 10.1016/j.virusres.2017.07.004. PubMed PMID: 28698046; PMCID: PMC7114558. 28698046 PMC7114558

