# Supplementary material for: Differential CD4+ T-Cell Cytokine and Cytotoxic Responses Between Reactivation and Latent Phases of Herpes Zoster Infection
Source: Pathog Immun. 2023 Feb 17;7(2):171–88. doi: 10.20411/pai.v7i2.560 (PMC9973729; doi:10.20411/pai.v7i2.560)
Supplement: Supplemental Figures 1–6 [file pai-7-171-s01.pdf]

PATHOGENS AND IMMUNITY
IN ASSOCIATION WITH CASE WESTERN RESERVE UNIVERSITY AND THE RICHARD J. FASENMYER FOUNDATION

SUPPLEMENTAL FIGURES

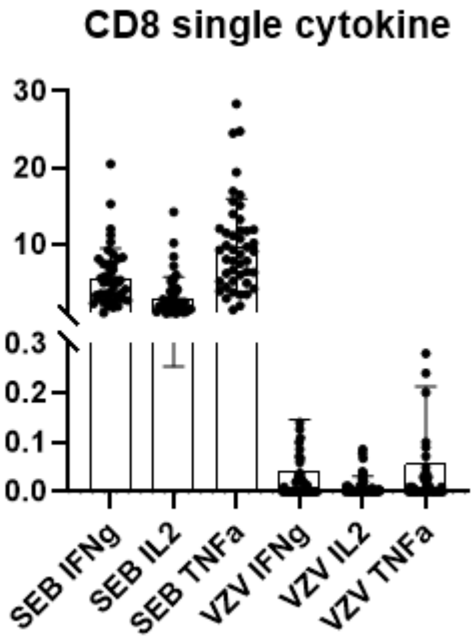

Supplementary Figure 1. Minimum T-cell cytokine response under VZV stimulation for CD8+ total memory cells. Each dot represents a unique individual.

Jin W, Fang M, Sayin I, Smith C, Hunter JL, Richardson B, Golden JB, Haley C, Schmader KE, Betts MR, Tyring SK, Cameron CM, Cameron MJ, Canaday DH. Differential CD4+ T-Cell Cytokine and Cytotoxic Responses Between Reactivation and Latent Phases of Herpes Zoster Infection. *Pathogens and Immunity*. 2023;7(2):171–188. doi: 10.20411/pai.v7i2.560.

PATHOGENS AND IMMUNITY
In Association with Case Western Reserve University and The Richard J. Fasenmyer Foundation

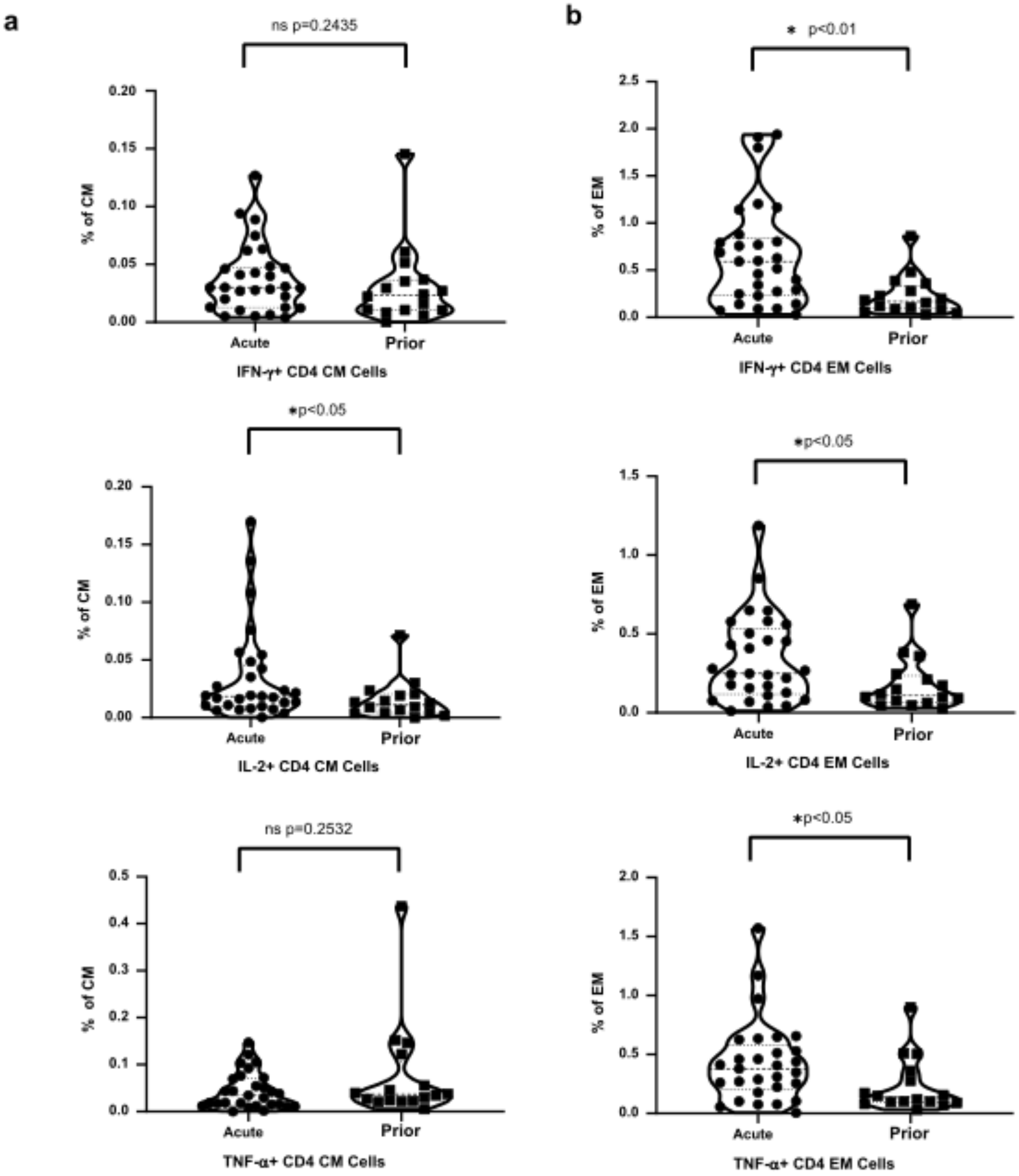

Supplement Figure 2. Violin plot for cytokine positive T cells percentages under VZV stimulation in a) central memory (CM) and b) effector memory (EM) CD4+ T cells. Net positive (VZV stim - no stim control) displayed as % positive with each dot as a unique individual.

Jin W, Fang M, Sayin I, Smith C, Hunter JL, Richardson B, Golden JB, Haley C, Schmader KE, Betts MR, Tyring SK, Cameron CM, Cameron MJ, Canaday DH. Differential CD4+ T-Cell Cytokine and Cytotoxic Responses Between Reactivation and Latent Phases of Herpes Zoster Infection. *Pathogens and Immunity*. 2023;7(2):171–188. doi: 10.20411/pai.v7i2.560.

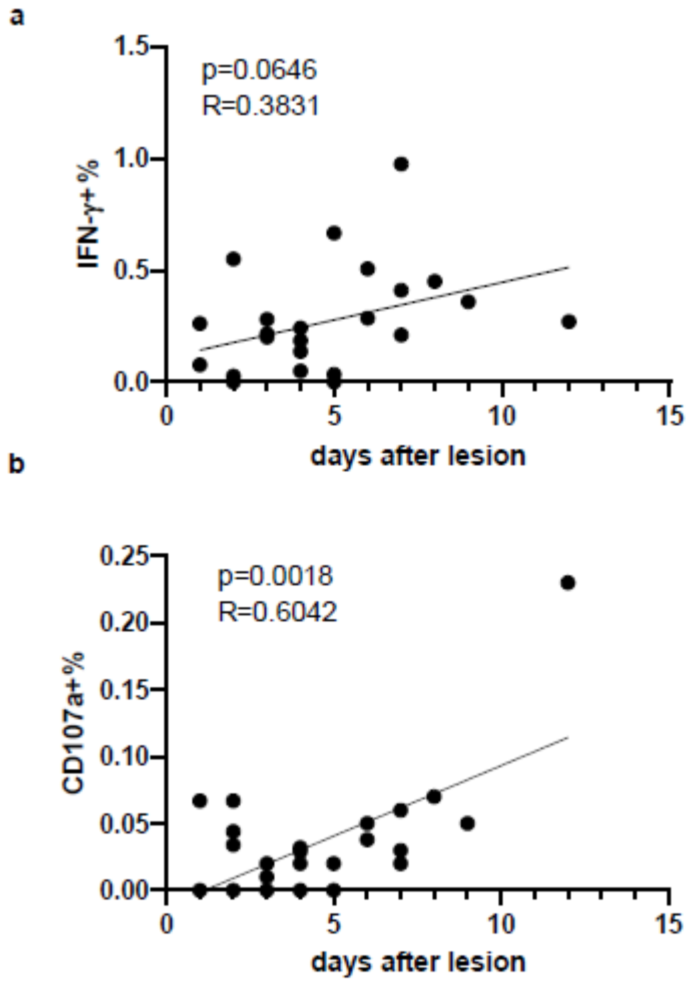

Supplementary Figure 3. Correlation in acute HZ individuals between days since lesions began and % expression of a) IFN-γ% or b) CD107a% by total memory CD4+ T cells after in vitro stimulation with VZV-antigen. Each dot represents a unique individual.

Jin W, Fang M, Sayin I, Smith C, Hunter JL, Richardson B, Golden JB, Haley C, Schmader KE, Betts MR, Tyring SK, Cameron CM, Cameron MJ, Canaday DH. Differential CD4+ T-Cell Cytokine and Cytotoxic Responses Between Reactivation and Latent Phases of Herpes Zoster Infection. *Pathogens and Immunity*. 2023;7(2):171–188. doi: 10.20411/pai.v7i2.560.

PATHOGENS AND IMMUNITY
IN ASSOCIATION WITH CASE WESTERN RESERVE UNIVERSITY AND THE RICHARD J. FASENMYER FOUNDATION

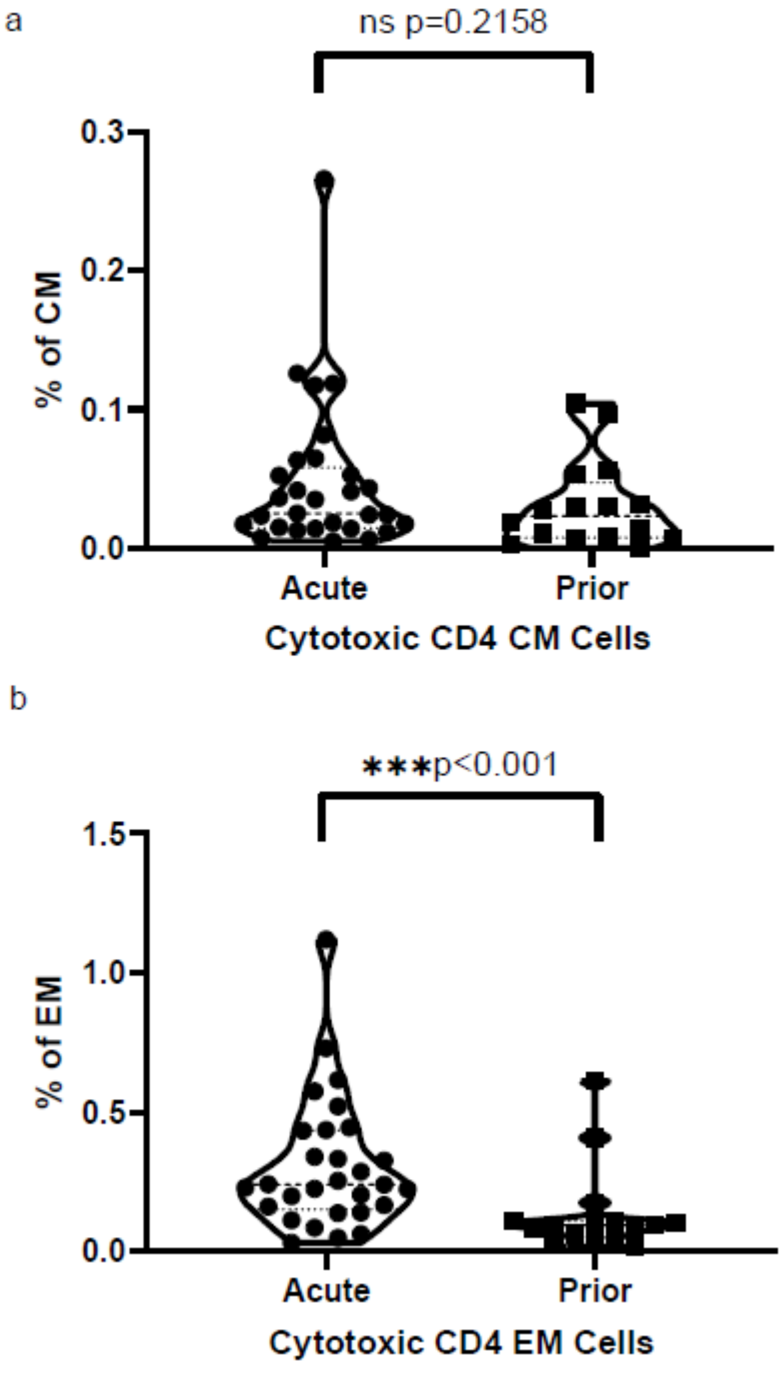

Supplementary figure 4. Violin plots for cytotoxic CD4+ T cells percentages in a) central memory b) effector memory CD4+ T cells after VZV stimulation cytotoxic CD4+T cell percentage in total memory CD4+ T cells. Each dot represents a unique individual.

Jin W, Fang M, Sayin I, Smith C, Hunter JL, Richardson B, Golden JB, Haley C, Schmader KE, Betts MR, Tyring SK, Cameron CM, Cameron MJ, Canaday DH. Differential CD4+ T-Cell Cytokine and Cytotoxic Responses Between Reactivation and Latent Phases of Herpes Zoster Infection. *Pathogens and Immunity*. 2023;7(2):171–188. doi: 10.20411/pai.v7i2.560.

PATHOGENS AND IMMUNITY
IN ASSOCIATION WITH CASE WESTERN RESERVE UNIVERSITY AND THE RICHARD J. FASENMYER FOUNDATION

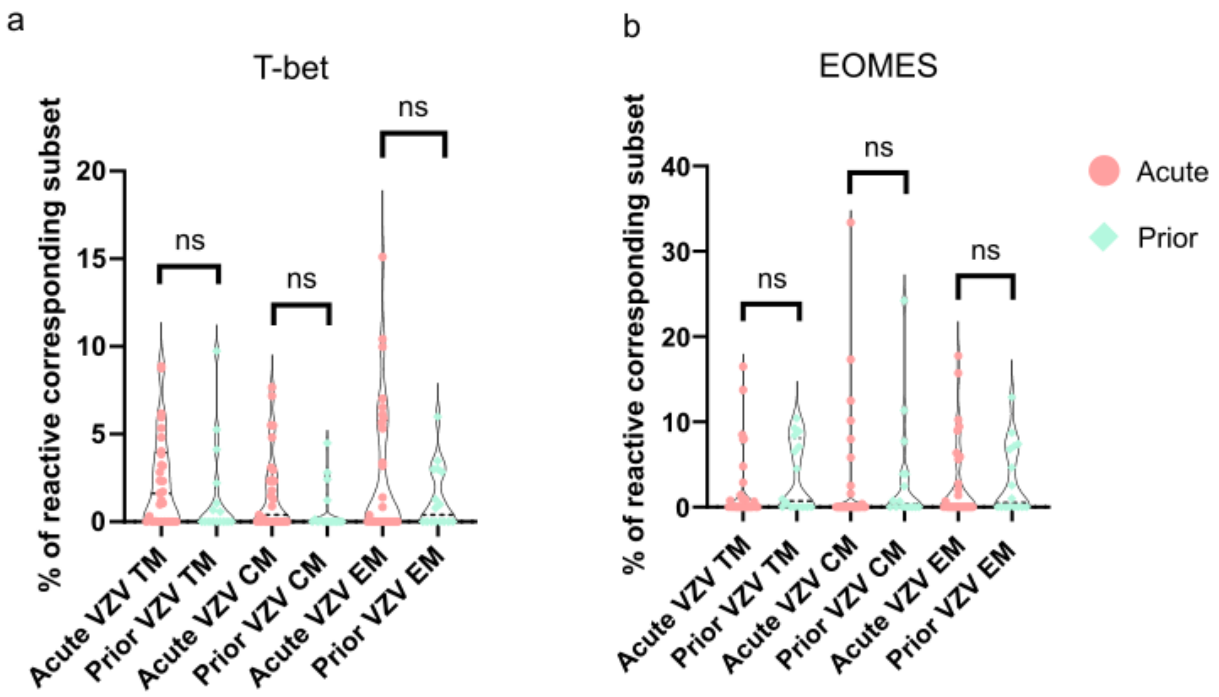

Supplementary Figure 5. T-bet and EOMES expression in different reactive CD4+ T-cell memory subsets under different stimulation for donors in different VZV history groups a) T-bet expression in reactive total memory (TM), central memory (CM) and effector memory (EM) cells b) EOMES expression in reactive total memory (TM), central memory (CM) and effector memory (EM) cells. Each dot represents a unique individual. (red dot: acute HZ group; green dot: prior HZ group).

Jin W, Fang M, Sayin I, Smith C, Hunter JL, Richardson B, Golden JB, Haley C, Schmader KE, Betts MR, Tyring SK, Cameron CM, Cameron MJ, Canaday DH. Differential CD4+ T-Cell Cytokine and Cytotoxic Responses Between Reactivation and Latent Phases of Herpes Zoster Infection. *Pathogens and Immunity*. 2023;7(2):171–188. doi: 10.20411/pai.v7i2.560.

PATHOGENS AND IMMUNITY
IN ASSOCIATION WITH CASE WESTERN RESERVE UNIVERSITY AND THE RICHARD J. FASENMYER FOUNDATION

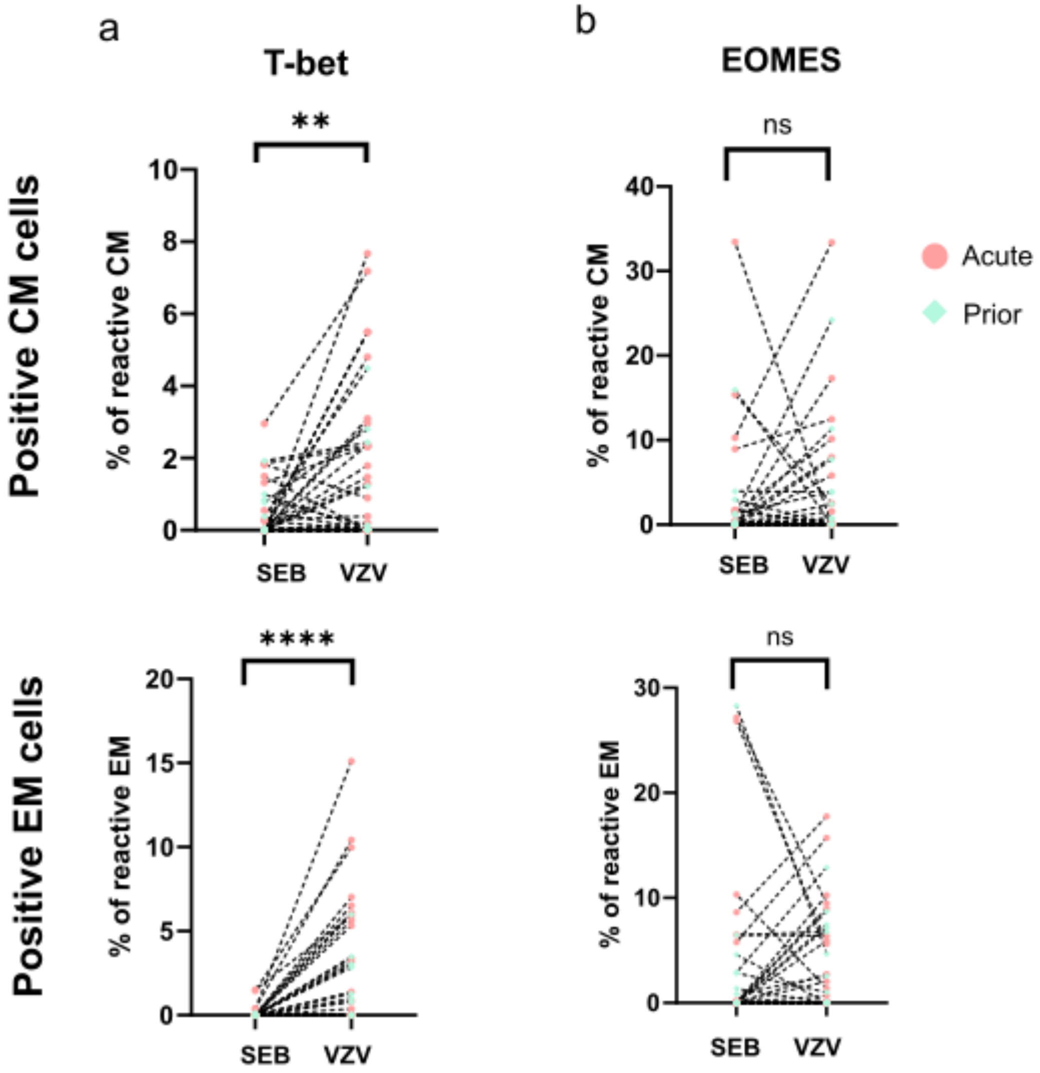

Supplementary Figure 6. T-bet and EOMES expression in different reactive CD4+ T-cell memory subsets under different stimulation a) T-bet expression in reactive central memory (CM) and effector memory (EM) cells b) EOMES expression in reactive central memory (CM) and effector memory (EM) cells. Each dot represents a unique individual. Dotted line connected data points are from the same individual. (red dot: acute HZ group; green dot: prior HZ group).

Jin W, Fang M, Sayin I, Smith C, Hunter JL, Richardson B, Golden JB, Haley C, Schmader KE, Betts MR, Tyring SK, Cameron CM, Cameron MJ, Canaday DH. Differential CD4+ T-Cell Cytokine and Cytotoxic Responses Between Reactivation and Latent Phases of Herpes Zoster Infection. *Pathogens and Immunity*. 2023;7(2):171–188. doi: 10.20411/pai.v7i2.560.
